# Developing and Evaluating Guidelines to Prevent Overdependence on Digital Therapeutics in Children and Adolescents: Randomized Controlled Trial

**DOI:** 10.2196/69248

**Published:** 2025-12-24

**Authors:** Euno Kim, Hajae Jeon, Junghan Lee, Hyangkyeong Oh, Meelim Kim, Jaeyong Shin, Eunjoo Kim

**Affiliations:** 1 College of Nursing Yonsei University Seoul Republic of Korea; 2 Department of Public Health Graduate School Yonsei University Seoul Republic of Korea; 3 Institute for Innovation in Digital Healthcare, Yonsei University Seoul Republic of Korea; 4 Division of Child and Adolescent Psychiatry, Department of Psychiatry and Institute of Behavioral Science in Medicine Severance Children’s Hospital, Yonsei University College of Medicine Seoul Republic of Korea; 5 Department of Convergence Medicine, Yonsei University College of Medicine Seoul Republic of Korea; 6 Institute of Behavioral Science in Medicine Yonsei University College of Medicine Seoul Republic of Korea; 7 Herbert Wertheim School of Public Health and Human Longevity Science, University of California San Diego, CA United States; 8 The Qualcomm Institute, University of California San Diego, CA United States; 9 The Design Lab, University of California San Diego, CA United States; 10 Department of Preventive Medicine Yonsei University College of Medicine Seoul Republic of Korea; 11 Department of Psychiatry Gangnam Severance Hospital, Yonsei University College of Medicine Seoul Republic of Korea

**Keywords:** digital therapeutics, overdependence prevention, children and adolescents, guideline development, mobile phone

## Abstract

**Background:**

Digital therapeutics (DTx) for children and adolescents with mental health problems have been developed in the health care industry. Despite reports of side effects from DTx for children and adolescents, there have been no guidelines to address the prevention of DTx overdependence among young users.

**Objective:**

This study aimed to identify the requirements for guidelines to prevent DTx overdependence in children and adolescents and to develop and evaluate these guidelines.

**Methods:**

We conducted 2 phases. This study first involved a phase I survey to develop guidelines, including assessments of smartphone usage and mental health conditions. The second phase evaluated the guidelines’ effectiveness, reliability, necessity, and satisfaction using a visual analog scale through a randomized controlled trial. Participants—45 children and adolescents aged 9-16 years and 42 caregivers—were randomly assigned to the experimental and control groups.

**Results:**

Phase I revealed that blocking mobile applications and notifications (mean 8.5, SD 1.8) and parental monitoring (mean 8.5, SD 2.1) were effective preventive features. Caregivers, children, and adolescents expressed concerns about the side effects and overdependence of DTx and decreased effects due to nonindividualized guidelines in subjective responses to the phase I survey. Based on these insights, personalized guidelines for phase II were developed, in which overall mean visual analog scale scores for guideline evaluation were higher in the experimental group, except for necessity among caregivers (mean 8.5, SD 1.3 versus mean 8.7, SD 1.2).

**Conclusions:**

Both caregivers and children and adolescents demonstrated the need for guidelines to prevent overdependence on DTx distinct from smartphone usage. Tailored guidelines may be acceptable for use in real-world therapeutic protocols. Guidelines to prevent overdependence on DTx in children and adolescents and to achieve a balance between their benefits and risks need to be established.

**Trial Registration:**

Clinical Research Information Service (CRiS) of the Republic of Korea KCT0008893; https://cris.nih.go.kr/cris/search/detailSearch.do?seq=25609

## Introduction

Digital therapeutics (DTx) are software-based interventions that use digital devices such as smartphones and computers to prevent, manage, and treat health conditions, including mental health problems in children and adolescents [1-3]. They improve accessibility, transcending temporal and spatial limitations, and provide personalized treatment experiences [2,4]. DTx products should be reviewed for their effectiveness and risks based on clinical evidence and approval from regulatory authorities (eg, the US Food and Drug Administration) [5]. Several DTx designed for children and adolescents use game-based elements to enhance engagement and therapeutic effectiveness [6,7].

Gamification, incorporating elements such as achievable challenges, immediate rewards, and personalization, shows promise in enhancing user engagement in digital mental health interventions [8]. A review of 7 products tailored for young individuals with mental health challenges (ie, EndeavorRx [Akili Interactive Labs, Inc], ATENTIVmynd [BrainFutures], RECOGNeyes [University of Nottingham], REThink [Babeș-Bolyai University, PsyTechs Research and Innovation Center], Mightier [Boston Children’s Hospital and Harvard Medical School Teaching Hospital], MindLight [PlayNice LLC], and SPARX [University of Auckland]) [6] found that despite using gamification strategies as interventions that could lead to overdependence, only 3 products (EndeavorRx, ATENTIVmynd, and SPARX) reported specific side effects in their clinical trials. Commonly reported side effects included headache, eye strain, and emotional reactions [9-11]. These side effects may negatively impact children and adolescents, who are in the most active growth phase of their lives. Although DTx are noninvasive and their side effects are relatively minor, they are still medical interventions applied to vulnerable populations. Thus, it is concerning that only 3 of 7 products explicitly addressed their side effects. A systematic literature review on DTx children with attention-deficit/hyperactivity disorder also found that most studies did not report safety outcomes, suggesting the need to identify its potential side effects and adverse effects [3]. In South Korea, during this research, DTx targeting children and adolescents were in the developmental phase, and no approved products were available.

Additionally, excessive screen time in children and adolescents, including the use of digital devices such as smartphones, televisions, and computers, may negatively affect their cognitive and socioemotional development [12,13]. The average daily screen time among children and adolescents aged 6 to 14 years is 2.77 hours [14], exceeding the AAP’s (American Academy of Pediatrics) recommended limit. Notably, this issue is of increasing importance given the rise in screen time among children and adolescents since the COVID-19 pandemic [14]. Given its association with mental health issues—such as obesity, sleep disturbances, depression, and anxiety—and its negative impact on parent-child interactions [12,15,16], to ensure the safe use of DTx for mental health in children and adolescents, it is essential to establish guidelines that prevent overdependence [17].

Although DTx may cause side effects ranging from mild to severe, and children and adolescents are particularly vulnerable when using digital devices, no specialized guidelines exist to address DTx overdependence. Government and organizational publications concerning media use in children and adolescents highlight safe practices for general digital engagement. However, given that DTx are used as therapeutic interventions, they necessitate a separate set of preventive measures to account for potential risks beyond those captured by conventional media-use guidelines. Thus, this study sought to define the requirements for such strategies and to evaluate their effectiveness, necessity, reliability, and satisfaction.

## Methods

### Study Design

**Figure 1:**
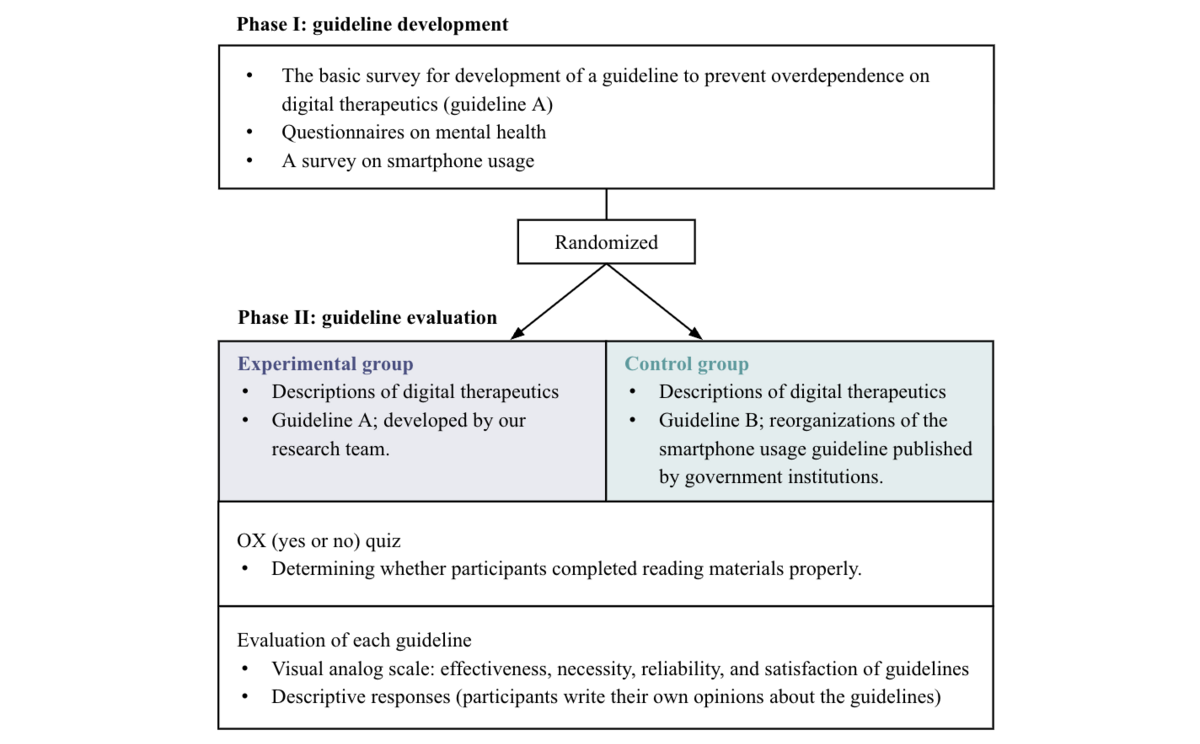
Study protocol of a 2-phase design for developing and evaluating guidelines to prevent overdependence on DTx among children and adolescents in South Korea. DTx: digital therapeutics; OX: yes or no.

This study was conducted in 2 phases: guideline development (phase I) and guideline evaluation (phase II). During phase I, participants responded to a basic survey designed to develop the guidelines to prevent overdependence on DTx. They also completed a survey on smartphone usage and a questionnaire on mental health. Randomization was performed after phase I using a random number table, assigning participants to either the experimental or control group in a 1:1 ratio. In phase II, participants were asked to complete an online survey that included detailed descriptions of DTx and guidelines for each assigned group. They also subsequently undertook an OX quiz (O=yes, X=no) to assess whether they had read the materials appropriately (Figure 1).

### Participants

A total of 87 participants were recruited between June and October 2023 at 2 tertiary hospitals, Severance Children’s Hospital and Gangnam Severance Hospital, in Seoul, South Korea. The participants included children and adolescents along with their caregivers, who visited outpatient clinics for mental health problems. Recruitment was conducted based on the recommendations of the doctors and after obtaining informed consent. Children and adolescents aged between 9 and 16 years, able to use their own smartphones, and who had permission from their caregivers were eligible to participate in this study. Individuals unable to complete the surveys independently or with mental health conditions that could interfere with study participation were excluded.

### Measurements

In phase I, a basic survey (phase I survey) was conducted with both caregivers and children and adolescents to inform the development of guidelines to prevent overdependence on DTx. Caregivers responded to a total of 16 items regarding DTx and how to manage their child’s smartphone usage. The survey used a combination of descriptive responses, multiple choice responses, and visual analog scales (VAS; range 0 to 10, 0=not at all, 10=extremely).

The following questions were answered descriptively: efforts to prevent the child from smartphone addiction (eg, “If there are any efforts you are currently making to prevent your child’s smartphone addiction, please describe them.”), acceptable daily DTx usage time (eg, “If your child is prescribed DTx, how many minutes/hours per day do you think is an appropriate amount of time to use it?”), features that may contribute to DTx overdependence (eg, “When your child uses DTx, what features do you think might lead to overdependence?”), features that may help prevent DTx overdependence (eg, “If you have any suggestions for ways to help prevent your child from becoming overly dependent on DTx, please describe them.”), and opinions on guideline-based DTx (eg, “If your child were to use DTx given guidelines to prevent overdependence on DTx, what would be the most looking forward to (potential advantages) and the most concerning (potential disadvantages)?”, “If there are concerns, what features would you like to see to support them (expectation of guidelines)?”). The question regarding the acceptable duration for DTx intervention was answered using a 4-option multiple-choice (eg, “What would be an acceptable duration for DTx intervention?”). The following questions were answered using VAS: features that may contribute to DTx overdependence (eg, communication, educational videos, and gamification), features that may help prevent DTx overdependence (eg, blocking mobile applications or game-based applications, and shutdown), and anticipated effectiveness of guideline-based DTx (eg, “How helpful do you think DTx given guidelines will be in managing your child’s symptoms?”).

Children and adolescents responded to a total of 3 items using descriptive responses and a VAS (range 0 to 10, where 0=not at all, and 10=extremely). Opinions on guideline-based DTx were collected through descriptive responses (eg, “If you use DTx given guidelines to prevent overdependence on DTx, what would be the most looking forward to (potential advantages) and the most concerning (potential disadvantages)?”). Perceived effectiveness of guideline-based DTx was assessed using the VAS (eg, “How helpful do you think DTx given guidelines will be in managing your symptoms?”).

The questionnaires on mental health were also conducted to assess participants’ baseline mental health status in phase I. Caregivers completed 3 questionnaires (ie, Smartphone Addiction Scale [SAS], Internet Gaming Use-Elicited Symptom Screen [IGUESS], and Children’s Behavior Checklist for Ages 6-18), while children and adolescents completed 8 questionnaires (ie, SAS, IGUESS, Patient health Questionnaire 9-Items [PHQ-9], Generalized Anxiety Disorder 7-Items [GAD-7], Perceived Stress Scale, Brief Fear of Negative Evaluation Scale [BFNE], Difficulties in Emotion Regulation Scale - Short Form, and Family Communication Scale [FCS]).

SAS is a screening tool derived from the Korea Youth Risk Behavior Web-Based Surveys self-reporting scale, consisting of 10 items with a total of 40 points. Responses were measured on a 4-point Likert scale, with scores determining addiction levels [18]. IGUESS is a 9-item survey to screen the risk of internet gaming disorder, in the fifth edition of the *DSM-5* (*Diagnostic and Statistical Manual of Mental Disorders* [Fifth Edition]). A score of 10 or above is indicative of a positive diagnosis of internet gaming disorder [19]. Children’s Behavior Checklist for Ages 6-18 is a component of the Achenbach System of Empirically Based Assessment. It is administered by caregivers and is used to identify behavioral and emotional issues in children and adolescents aged 6 to 18 years [20].

PHQ-9 is a brief instrument for assessing the severity of depression, completed by patients. PHQ-9 consists of 9 items, each rated on a 4-point Likert scale [21]. GAD-7 is a preliminary screening instrument comprising 7 items, designed to identify the presence of anxiety disorders. GAD-7 uses a 4-point Likert scale for rating [22]. The Perceived Stress Scale is a classic instrument for assessing perceived stress, containing 10 items. It is rated on a 5-point Likert scale [23]. BFNE is a widely used instrument aimed at assessing an individual’s tolerance for the possibility that they might be judged disparagingly or hostilely by others. BFNE consists of 12 items, and respondents provide their answers using a 5-point Likert scale [24]. Difficulties in Emotion Regulation Scale - Short Form is an 18-item self-report questionnaire, measuring difficulties in emotion regulation. Each of these items is evaluated using a 5-point Likert scale [25]. FCS is a component included in the Family Adaptability and Cohesion Evaluation Scale IV. FCS comprises 10 items designed to measure the degree of positive communication among family members. Each of these items is evaluated using a 5-point Likert scale [26].

In phase II, guidelines were evaluated using a VAS (range 0 to 10, 0=not at all, 10=extremely), covering 4 aspects: effectiveness, necessity, reliability, and satisfaction (Table S1 in Multimedia Appendix 1).

### Interventions

The intervention of this study was guidelines to prevent overdependence on DTx (guideline A), developed by our research team based on the findings of phase I, reflecting the needs of potential users of DTx for children and adolescents. These guidelines were also organized according to research that explored the potential challenges of digital interventions for children and adolescents, such as ethical issues, safety from side effects and privacy, and interpersonal relationships of family or caregivers [27,28]. Additionally, development was informed by publications related to media use and the Family Media Plan issued by the AAP.

The primary focus of this development was to enable users to create practical action plans using the guidelines. Guideline A comprised four sections: (1) check, (2) plan, (3) action, and (4) smart (Note S1 in Multimedia Appendix 1).

The check section starts with the assessment of the digital dependency of children and adolescents through SAS and IGUESS. It also includes information on the negative symptoms that may arise from DTx overuse. This section provides users with information on identifying potential side effects and precautions, addressing concerns raised in the phase I survey.

The plan section helps users develop personalized strategies for DTx, allowing them to specify the tools or devices needed for treatment and their usage (eg, purpose, duration, and frequency). This section reflects the need for individualized guidelines based on differences in diagnosis and prescription, as identified in the participants’ requirements in the phase I survey. Plans differ depending on the child’s age, with children ≤12 developing plans with caregivers, and adolescents aged 13 years and older developing their own plans.

The action section includes specific behavioral actions to perform in real life to prevent overdependence on DTx. It addresses concerns such as increased exposure to digital devices and conflicts with family members due to DTx usage. Caregivers and children, and adolescents can discuss when and where they use DTx (eg, storage location and usage time) and plan activities to balance their online and offline lives (eg, daily physical activities and DTx free time). This content refers to the recommendations for physical activity among children and adolescents from the World Health Organization and AAP.

Lastly, the smart section describes the use of functions or mobile applications that can help prevent overdependence on DTx. This section also includes regular contact with professionals, reflecting the participants’ need to receive ongoing and timely specialist feedback, as identified in the phase I survey. Guideline A was reviewed by a panel of professional experts, including 2 pediatric psychiatrists (EK and JL) and digital health care professionals (MK and JS).

In phase II, guideline A was provided to the experimental group, while the control group received a reorganized version of general smartphone usage guidelines (guideline B) published by government institutions in South Korea, serving as treatment as usual (Note S1 in Multimedia Appendix 1) [29,30]. Each guideline was tailored to different user groups: caregivers, children (aged 6-12 years), and adolescents (aged 13-18 years).

### Statistical Analysis

The sample size was determined to detect a clinically meaningful difference in mean outcomes between the experimental group, which received a tailored guideline for DTx (guideline A), and the control group, which received a conventional smartphone use guideline (guideline B). Using G*Power (Heinrich-Heine-Universität Düsseldorf) software, a total sample size of 52 participants was calculated based on an effect size of 0.8, a 2-tailed significance level of 5%, and a statistical power of 80%. To account for an anticipated dropout rate of 10%-15%, the required minimum sample size was adjusted to at least 60 participants. A total of 87 and 50 participants (owing to missed follow-ups after phase I) were included in the phase I and phase II analyses, respectively.

All statistical analyses were conducted using Jupyter Notebook (version 6.5.4). Descriptive statistics were used to summarize questionnaire responses, survey data, and other quantitative outcomes. Differences in baseline characteristics were analyzed using independent-samples *t* tests for continuous variables and chi-square tests for categorical variables. Mann-Whitney *U* tests were used to analyze nonparametric outcome variables in phase II. Thematic analysis was used to analyze the qualitative data from the phase I survey and the evaluation of guidelines in phase II. The participants’ responses to the questionnaire were used as received, without any modifications.

### Ethical Considerations

This study was approved by the institutional review board (IRB) at Severance Children’s Hospital (IRB No. 4-2023-0366) and Gangnam Severance Hospital (IRB No. 3-2023-0129) and registered with the Clinical Research Information Service (KCT0008893). Written informed consent was obtained from all participants. To accommodate age-related differences in comprehension, consent materials were developed in 3 versions: for caregivers, for children (aged 9-13 years), and for adolescents (aged 13-16 years). All data collected were fully deidentified and securely stored to maintain participant privacy and confidentiality. Participants were compensated with a gift card after completing all surveys (Korean won 30,000 [US $30] for each phase).

## Results

### Overview

**Figure 2:**
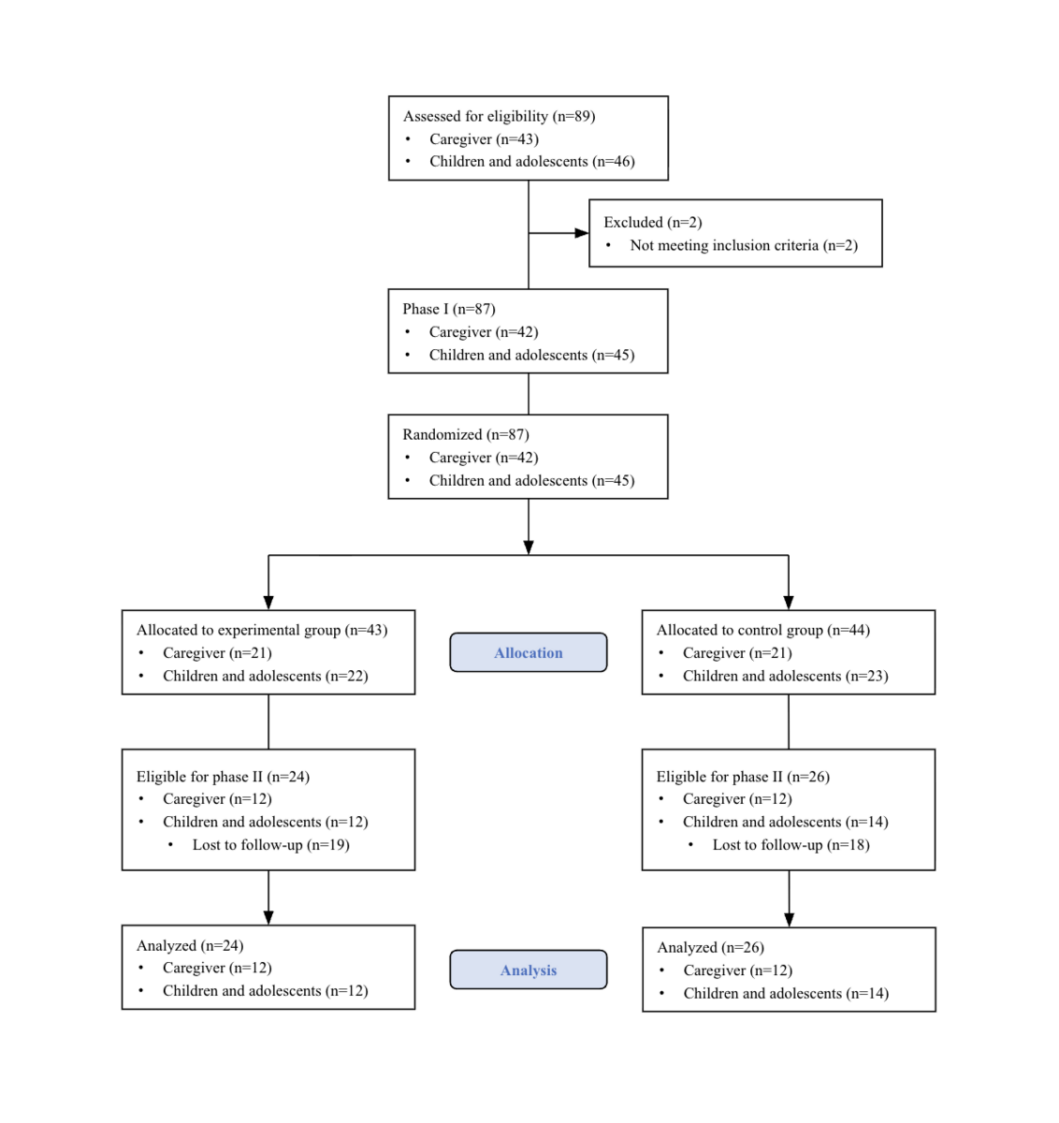
CONSORT flow diagram for this study. CONSORT: Consolidated Standards of Reporting Trials.

The flow of participants from recruitment to analysis is shown in Figure 2. There were no substantial differences in the baseline characteristics between the groups. Table 1 shows the detailed baseline characteristics of each caregiver and children and adolescents. In total, 24 caregivers (age: mean 46.4, SD 5.5 years) and 26 children and adolescents (age: mean 12.3, SD 1.9 years) completed phase II. Figure S1 in Multimedia Appendix 1 presents the results of smartphone usage among children and adolescents.

**Table 1:** Baseline characteristics of caregivers and children and adolescents in both the experimental and control groups.

Variable					Experimental caregivers (n=21) and children and adolescents (n=22)		Control caregivers (n=21) and children and adolescents (n=23)
**Caregivers’ characteristics**							
	**Gender, n (%)**						
		Male	0 (0)			1 (4.8)	
		Female	21 (100)			20 (95.2)	
	Age (years), mean (SD)			46 (5.6)			46.7 (5.5)
**Characteristics of children and adolescents**							
	**Gender, n (%)**						
		Male	13 (59.1)			17 (73.9)	
		Female	9 (40.9)			6 (26.1)	
	Age (years), mean (SD)			12.5 (1.9)			12.0 (1.8)
	**Education, n (%)**						
		Elementary school	8 (36.4)			13 (56.5)	
		Middle school	11 (50)			6 (26.1)	
		High school	3 (13.6)			4 (17.4)	
**Diagnosis of mental illness^a^, n (%)**							
	Anxiety			4 (13.3)			3 (13)
	Asperger disorder			0 (0)			1 (4.3)
	Attention-deficit disorder			0 (0)			1 (4.3)
	Attention-deficit/hyperactivity disorder			17 (56.7)			8 (34.8)
	Autism spectrum disorder			1 (3.3)			0 (0)
	Delayed development			1 (3.3)			0 (0)
	Depression			2 (6.7)			1 (4.3)
	Dyslexia			0 (0)			1 (4.3)
	Obsessive-compulsive disorder			1 (3.3)			1 (4.3)
	Oppositional defiant disorder			1 (3.3)			0 (0)
	Panic disorder			0 (0)			1 (4.3)
	Posttraumatic stress disorder			0 (0)			1 (4.3)
	Tic disorders			1 (3.3)			2 (8.7)
	Tourette syndrome			2 (6.7)			2 (8.7)
	Not yet			0 (0)			1 (4.3)
**Resident family members, n (%)**							
	Three			7 (33.3)			7 (33.3)
	Four			12 (57.1)			14 (66.7)
	Five			2 (9.5)			0 (0)
**Children’s and adolescents**’ **smartphone usage time (hours), mean (SD)**							
	**Parental report**						
		Weekdays	3.7 (2.8)			3.1 (2.2)	
		Weekends	5.3 (3.1)			4.0 (2.1)	
	**Self-report**						
		Weekdays	3.1 (1.9)			2.4 (1.7)	
		Weekends	4.4 (2.7)			3.3 (1.7)	
**Scale or questionnaire (score), mean (SD)**							
	Caregivers			—^b^			—
	Smartphone Addiction Scale^c^			24.2 (8.7)			22.3 (5.8)
	Internet Gaming Use-Elicited Symptom Screen^c^			9.7 (9.1)			3.6 (4.6)
	**Children’s Behavior Checklist for ages 6-18 years, n (%)**						
		Total problems score	66.4 (10.7)			59.1 (12.8)	
		Internalization score	65.0 (13.0)			58.8 (11.9)	
		Externalization score	59.1 (18.6)			56.7 (15.1)	
**Children and adolescents, mean (SD)**							
	Smartphone Addiction Scale			18.9 (5.6)			18.7 (5.8)
	Internet Gaming Use-Elicited Symptom Screen			6.5 (4.3)			5.7 (3.3)
	Patient Health Questionnaire			8.7 (7.0)			7.2 (5.3)
	Generalized Anxiety Disorder			7.2 (5.5)			6.1 (6.8)
	Perceived Stress Scale			20.2 (4.7)			21.3 (4.0)
	Difficulties in Emotion Regulation Scale - Short Form			48.5 (13.8)			45.6 (15.1)
	Brief Fear of Negative Evaluation Scale			41.0 (9.5)			40.7 (12.2)
	Family Communication Scale			33.2 (8.6)			36.5 (8.9)

^a^Both principal and secondary diagnoses were included.

^b^Not applicable.

^c^These scale results reflect parental evaluations based on daily observations of their children.

#### Basic Survey for the Development of Guideline A (Phase I Survey)

Using the VAS, caregivers assessed features that may contribute to or may help prevent overdependence on DTx. According to Table 2, gamification showed the highest score (mean 6.5, SD 2.6) as potential overdependence inducers in the DTx, followed by communication (mean 6.4, SD 2.6) and educational videos (mean 4.1, SD 2.6). The most effective features for overdependence prevention were blocking mobile applications or notifications (mean 8.5, SD 1.8), parental monitoring (mean 8.5, SD 2.1), and shutdown (mean 8.2, SD 1.7). Blocking mobile applications or notifications is a function to prevent access to other applications or notifications while using DTx. Parental monitoring refers to a function that allows caregivers to monitor their children’s use of DTx; shutdown refers to a function that automatically switches the device off after a set DTx treatment time.

**Table 2:** Results of the basic survey for the development of guideline A (phase I survey) in caregivers and children or adolescents.

Outcome measures		Caregivers (n=42)		Children and adolescents (n=45)		
**DTx^a^ features that may contribute to DTx overdependence (VAS^b^), mean (SD)**						
	Communication		6.4 (2.6)		—^c^	
	Educational videos		4.1 (2.6)		—	
	Gamification		6.5 (2.6)		—	
**DTx features that may help prevent DTx overdependence (VAS), mean (SD)**						
	Blocking mobile applications or notifications		8.5 (1.8)		—	
	Parental monitoring		8.5 (2.1)		—	
	Shut down		8.2 (1.7)		—	
	Anticipated effect of guidelines (VAS)		6.3 (1.6)		6.4 (1.8)	
	Acceptable daily usage time of DTx (min)		38.6 (23.2)		—	
**Acceptable duration for DTx intervention (weeks), n (%)**						
	4		11 (26.2)		—	
	8		9 (21.4)		—	
	12		16 (38.1)		—	
	16		6 (14.3)		—	

^a^DTx: digital therapeutics.

^b^VAS: visual analog scale.

^c^Not available.

Table 2 presents the anticipated effects of the guideline (mean 6.3, SD 1.6) among caregivers and children and adolescents (mean 6.4, SD 1.8). Caregivers considered 38.6 (SD 23.2) minutes as an acceptable daily average usage time for DTx. The most acceptable duration for the DTx intervention period was 12 weeks (16 individuals, 38.1%), followed by 4 weeks (11 individuals, 26.2%), 8 weeks (9 individuals, 21.4%), and 16 weeks (6 individuals, 14.3%).

Participants also provided descriptive responses regarding potential advantages, disadvantages, and expectations of guidelines to prevent DTx overdependence (see Table S3 in Multimedia Appendix 1 for details). Caregivers mentioned potential advantages such as preventing side effects and overdependence, enhancing children’s self-regulation, reducing caregivers’ anxiety, and improving motivation for treatment. Potential disadvantages mentioned were side effects and overdependence, increased exposure to digital devices, and decreased effectiveness of treatments due to nonindividualized guidelines. Additionally, caregivers wanted the guidelines to include parental monitoring, blocking other applications or notifications while using DTx, ongoing feedback from professionals, integration with nondigital treatments, and activities such as stretching or mental relief time.

Children and adolescents also mentioned potential advantages such as the prevention of side effects and overdependence, improved self-regulation, and increased reliance on DTx. However, potential disadvantages included side effects and overdependence, treatment failure due to nonindividualized or strict guidelines, and conflicts with family members in children and adolescents’ responses.

#### Primary Outcome: VAS Scores for Guideline Evaluation

The primary outcomes were VAS scores for guideline evaluation, focusing on effectiveness, necessity, reliability, and satisfaction. In the experimental group, 25 of the 43 (58.1%) participants completed the primary outcome measures, while 19 (44.2%) individuals did not complete this study. Similarly, in the control group, 26 of 44 (59.1%) participants completed these measures, while 18 (41%) participants did not.

**Figure 3:**
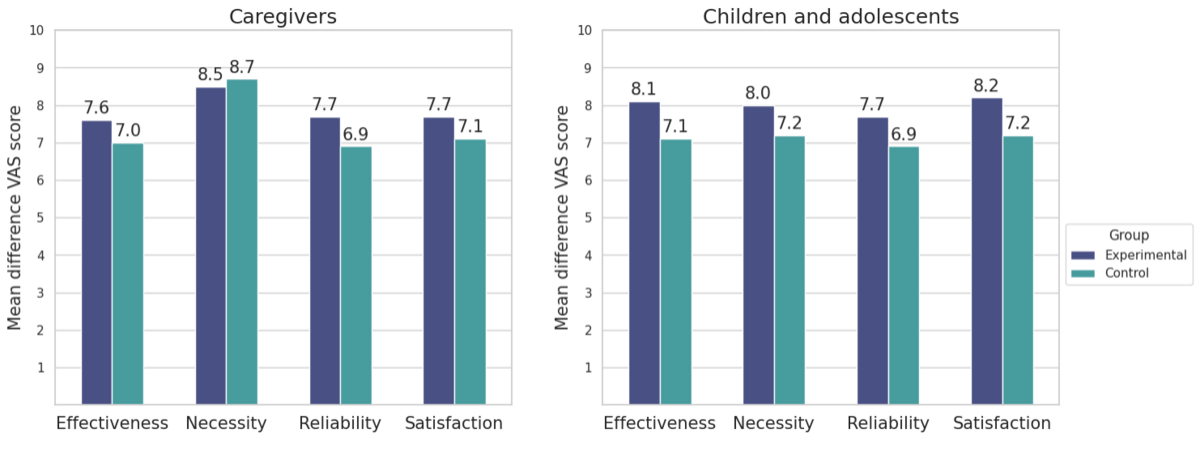
Comparison of mean VAS scores for guideline evaluation between experimental (guideline A) and control (guideline B) groups among caregivers and children and adolescents in phase II. Scores were based on perceived effectiveness, necessity, reliability, and satisfaction. VAS: visual analog scale.

According to Figure 3, the overall VAS scores were generally higher in the experimental group, except for necessity among caregivers, which was higher in the control group (mean 8.7, SD 1.2; mean 8.5, SD 1.3). For caregivers in the experimental group, the highest scores were for necessity, followed by similar scores for reliability and satisfaction (mean 7.7, SD 1.2 and mean 7.7, SD 1.1, respectively), and effectiveness (mean 7.6, SD 1.0). Caregivers in the control group also attributed the highest score to necessity (mean 8.7, SD 1.2), followed by satisfaction (mean 7.1, SD 1.4), effectiveness (mean 7.0, SD 1.6), and reliability (mean 6.9, SD 1.8). The largest difference was in the reliability scores, which showed a mean difference of 0.8 between the caregiver groups.

In the experimental group, satisfaction scored the highest (mean 8.2, SD 1.3), followed by effectiveness (mean 8.1, SD 1.6), necessity (mean 8.0, SD 2.1), and reliability (mean 7.7, SD 2.1). In the control group, children and adolescents rated necessity and satisfaction at 7.2 (SD 1.8) and 7.2 (SD 2.0), respectively, followed by effectiveness (mean 7.1, SD 2.1) and reliability (mean 6.9, SD 2.4). The largest differences among children and adolescents were noted in the effectiveness and satisfaction scores, with each showing a difference of 1.

#### Secondary Outcome: The Qualitative Data Analysis

In phase II, qualitative data were organized into four primary themes: (1) satisfaction, (2) effectiveness, (3) necessity, and (4) knowledge. Verbatim examples of each theme are presented in Table 3.

**Table 3:** Descriptive responses from caregivers and children, and adolescents in phase II regarding their evaluation of the assigned guidelines.

Themes and groups				Verbatim examples
**Caregivers**				
	**Satisfaction**			
		Control	“I expected more specific guidelines with details.”	
		Experimental	“It is more detailed than I expected, so I think it will be better than what I was worried about.” “It was good to plan things in advance with parents.” “It was good to have a prescription like common medicine. And it will be helpful to know how the treatment is progressing.”	
	**Effectiveness**			
		Control	“When introducing DTx using smart devices, it will be needed to try to minimize dependence on these devices. However, the given guidelines may feel like a general overview rather than specific guidance.”	
		Experimental	“I was worried about potential side effects when my child started treatment, but after reviewing the guidelines, it seems like I can be less concerned about DTx.”	
	**Necessity**			
		Control	“The provided guidelines seem just general guidelines for preventing overdependence on smartphones. I’m wondering why the name of this guideline is the prevention for ‘DTx’.”	
		Experimental	“Without the provided guidelines, it seems that the therapeutic objectives could be compromised.”	
	**Knowledge**			
		Control	“After using a smartphone, it is necessary to do eye exercises.”	
		Experimental	“DTx should be used only for duration according to the prescription.” “It is advisable to designate a specific location, among other considerations, when using digital therapy.” “There are useful apps for DTx can be utilized.”	
**Children and adolescents**				
	**Satisfaction**			
		Control	“It would be good to provide more detailed guidelines.”	
		Experimental	“Recommendations for using time control apps and commitments to physical activities.”	
	**Knowledge**			
		Control	“I became aware of how much I use my phone.” “I learned various rules that are necessary when using a smartphone.” “Things that can prevent eye fatigue and deterioration.” “Delete unnecessary apps.”	
		Experimental	“I learned that the DTx created for treatment purposes carry the risk of overdependence.” “I learned that excessive dependence can lead to conflicts with others.” “Don’t put digital devices near you when you sleep.” “I’ve come to know what DTx is.”	

Feedback on guideline A was more favorable compared to guideline B. Participants in the experimental group evaluated the guidelines more positively, using phrases such as “more detailed than I expected,” indicating a high level of satisfaction. Conversely, participants in the control group expressed a need for more detailed guidelines, with comments such as “would be good to have more detailed guidelines,” reflecting their relative dissatisfaction. Moreover, caregivers and children, and adolescents in the experimental group reported being satisfied with the contents of guideline A, such as planning with the caregiver, monitoring treatment progress, and recommendations for physical activities.

Responses regarding the effectiveness of the guidelines varied between the groups. A caregiver in the control group criticized the guidelines for being too generalized, stating that they “might feel like a general overview.” Contrastingly, a caregiver in the experimental group reported reduced anxiety related to the use of DTx, noting that “I can allow myself to be less concerned about DTx,” suggesting a positive perception of guideline A’s effectiveness.

The perception of necessity also differed markedly between the groups. A caregiver from the control group questioned the applicability of guideline B to DTx, commenting that it seemed more suited for general smartphone use than for specifically addressing DTx. Meanwhile, a caregiver in the experimental group emphasized that “the therapeutic objectives could be compromised without the provided guidelines,” indicating a strong perceived necessity for guideline A.

The knowledge gained from each guideline reveals a distinct focus. Participants in the experimental group highlighted learning about specific overdependence prevention contents, such as designating locations and understanding overdependence risks associated with DTx. By contrast, the control group focused more on general smartphone issues, such as eye problems and smartphone usage.

These themes illustrated the different levels of satisfaction, perceived effectiveness, necessity, and knowledge acquisition between the experimental and control groups, highlighting the impact of guideline specificity and relevance on user reception and learning outcomes.

## Discussion

### Principal Findings

This randomized trial comprised 2 phases. Phase I investigated the needs of potential users regarding guidelines to prevent overdependence on DTx among children and adolescents, to inform its development. In phase II, we evaluated the developed guidelines. As DTx administration differs fundamentally from general smartphone usage, the guidelines developed in this study are more viable than existing smartphone addiction guidelines. The co-design process used to develop these guidelines with actual users contributes to their acceptability among a range of potential users.

Phase I results showed that both caregivers and children and adolescents were concerned about side effects and overdependence on DTx, indicating the need for prevention guidelines for the children and adolescents population to ensure ethical use. Gamification was identified as the most addictive component among various DTx intervention elements for children and adolescents. Additionally, gamification may lead to privacy infringements and social overload [31]. Around 70% of DTx for children and adolescents (7 of 10 DTx products) in the United States and over 80% of them (5 of 6 DTx products) in South Korea use gamification as their core intervention component [32]. However, these products did not clearly address caregivers’ concerns regarding potential side effects and overdependence on DTx. Nonetheless, gamification is an effective strategy to encourage participation and enhance the effectiveness of digital intervention [33,34]. Given the ambivalence surrounding gamification, it is necessary to establish guidelines to prevent overdependence on DTx in children and adolescents, achieving a balance between their benefits and risks.

Furthermore, the phase I results indicated that DTx users would prefer developers to include overdependence prevention features, such as blocking other applications or notifications while using DTx, parental monitoring tools, and shutting down the application. Currently, some applications can restrict access to other applications using blockers such as Digital Wellbeing (Google LLC), Google Family Link (Google LLC), and AirDroid Parental Control for Androids (Sand Studio) [35], as well as features on Content and Privacy Restrictions (Apple Inc) for iPhones [36]. Although each operating system offers some third-party applications or features, there are currently no cases where DTx for children and adolescents has been tested to incorporate these features or functions into their intervention protocols. Our findings may help DTx developers include these features or functions in their systems to better reflect users’ concerns about DTx overdependence.

Along with overdependence prevention features for DTx developers, DTx users demonstrated a need for individualized and actionable guidelines to prevent DTx overdependence for children and adolescents, which can be implemented collaboratively by the family. To the best of our knowledge, however, there are no customized or practical guidelines to prevent overdependence on DTx. In order to address these needs and issues, we developed a guideline, which is called guideline A in this study, that includes individualized and actionable components to prevent DTx overdependence in children and adolescents. The guideline begins by assessing the individual level of digital dependency of the children and adolescents and the potential adverse effects of DTx in the check section. This establishes the rationale for developing individual strategies and behavioral actions in the following sections. The plan and action sections lead the users in adapting the guidelines to their individual contexts, including reviewing their prescribed DTx, appropriately storing devices when not to be used or during sleep, and maintaining a balance with offline activities. Finally, the smart section connects children and adolescents and their caregivers with health care professionals, ensuring the guideline not only engages family members but also invites health care professionals to continue monitoring the patient’s condition progression.

After the guideline development, we compared guideline A with an existing general-use guideline (guideline B). The effectiveness, reliability, and satisfaction of guideline A were higher among caregivers and children and adolescents than those of guideline B. This aligns with the results from the qualitative data. Guideline A showed more promising results than guideline B across all themes, including satisfaction, effectiveness, necessity, and knowledge. These findings are consistent with previous research showing that interventions targeting screen time reduction in children had statistically significant effects. A meta-analysis demonstrated that such interventions—often incorporating knowledge dissemination and increased physical activity—were effective in reducing screen time [37]. These components are also reflected in our guidelines, particularly in the plan and action sections, which emphasize education and promotion of offline activities. Given the consistent advantages demonstrated across both quantitative and qualitative measures, guideline A appears suitable for distribution in the DTx market and for integration into DTx protocols for children and adolescents.

Despite its strengths, this study has some limitations. First, the sample did not include individuals who had actually used DTx, given that there are no publicly available DTx for children and adolescents [38]. Only a few DTx for children and adolescents are available for specific populations in clinical trials as part of the regulatory process [6,39-42]. Since children and adolescents are the potential future users of DTx, we described a fictitious DTx when testing the validity of the developed guidelines. Second, although participants were recruited from multiple tertiary hospitals, the selection process involved physician referrals, and this study was conducted in a hospital-based setting. This purposive selection of study sites and participants may have introduced selection bias. In interpreting these findings, it is important to consider South Korea’s unique cultural and technological context. The country has one of the highest smartphone penetration rates among adolescents [43], and strong parental involvement in children’s digital usage is common [44]. Unlike Western countries that emphasize autonomy and privacy, South Korea tends to adopt a more centralized approach, with government and public institutions offering structured guidance on youth digital health practices [4,45-47]. These contextual factors may influence the acceptance and expectations of such interventions. Future studies should therefore test the effectiveness, necessity, reliability, and satisfaction of this guideline with actual DTx users for children and adolescents. Furthermore, future research should examine how these guidelines affect the development process of DTx developers and the therapeutic process of health care professionals. Future studies should also explore whether these guidelines are useful in developing reliable DTx for children and adolescents or beneficial in improving health outcomes for those who use DTx for children and adolescents.

### Conclusions

This is the first study to represent the development and testing of the guideline to prevent overdependence on DTx in children and adolescents. The results of this study provide insights into the concerns about DTx overdependence for children and adolescents, which inform the need for preventative guidelines regarding this issue. Additionally, this study provides evidence that the guideline which we developed for preventing DTx overdependence for children and adolescents may be acceptable to be used in therapeutic protocols in the real world. The impact of this guideline will not only be on the DTx users, but across the diverse health care settings and systems where DTx are used.

### Implications and Contributions

This study develops and evaluates guidelines to prevent overdependence on DTx in children and adolescents. The findings indicate that personalized guidelines may mitigate overuse concerns, and these guidelines are likely to be applicable in clinical settings, providing practical strategies for health care providers and caregivers.

## Data Availability

The datasets used or analyzed during this study are available from the corresponding author on reasonable request.

## Appendix

Multimedia Appendix 1 Tables S1-3, Figure S1, and Note S1.

Multimedia Appendix 2 CONSORT 2025 editable checklist.

## Abbreviations

AAP: American Academy of Pediatrics
BFNE: Brief Fear of Negative Evaluation Scale
DSM-5: Diagnostic and Statistical Manual of Mental Disorders (Fifth Edition)
DTx: digital therapeutics
FCS: Family Communication Scale
GAD-7: Generalized Anxiety Disorder 7-Items
IGUESS: Internet Gaming Use-Elicited Symptom Screen
IRB: institutional review board
PHQ-9: Patient Health Questionnaire 9-Items
SAS: Smartphone Addiction Scale
VAS: visual analog scale
