# Supplementary material for: Developing and Evaluating Guidelines to Prevent Overdependence on Digital Therapeutics in Children and Adolescents: Randomized Controlled Trial
Source: J Med Internet Res. 2025 Dec 24;27:e69248. doi: 10.2196/69248 (PMC12780698; doi:10.2196/69248)
Supplement: Multimedia Appendix 1 [file jmir_v27i1e69248_app1.pdf]

Table S1 in Multimedia Appendix 1. Summary of psychometric instruments used in the study.

Instrument	Purpose	Number of Items	Response Scale	Key Features
Patient Health Questionnaire-9 (PHQ-9)	Assessing severity of depression	9	4-point Likert scale	Brief self-report instrument completed by patients
Generalized Anxiety Disorder-7 (GAD-7)	Preliminary screening for anxiety disorders	7	4-point Likert scale	Designed to identify the presence of anxiety disorders
Perceived Stress Scale (PSS)	Assessing perceived stress	10	5-point Likert scale	Classic instrument for evaluating stress perception
Brief Fear of Negative Evaluation Scale (BFNE)	Assessing tolerance for being judged negatively by others	12	5-point Likert scale	Widely used measure for fear of negative evaluation
Difficulties in Emotion Regulation Scale – Short Form (DERS-SF)	Measuring difficulties in emotion regulation	18	5-point Likert scale	Self-report questionnaire, short form
Family Communication Scale (FCS)	Measuring positive communication among family members	10	5-point Likert scale	Subscale of FACES IV, focused on family communication
Visual Analogue Scale (VAS)	Evaluating guidelines (effectiveness, necessity, reliability, satisfaction)	-	0–10 scale (0 = not at all, 10 = extremely)	Used in Phase II guideline evaluation

Table S2 in Multimedia Appendix 1. OX (yes or no) quiz to determine whether participants completed reading guidelines properly in Phase II.

[1] Caregiver version

**Guideline A (Experimental group)**	**Guideline B (Control group)**
The following is information regarding the guidelines. Please read the sentences and mark O for correct and X for incorrect.	The following is information regarding the guidelines. Please read the sentences and mark O for correct and X for incorrect.
1. Overdependence on DTx can lead to negative symptoms such as spinal disorders, dry eyes, and obesity.	1. Excessive smartphone usage does not have a negative impact on mental health.
2. DTx should only be used for the prescribed duration in a designated location.	2. Excessive smartphone usage can lead to negative outcomes such as spinal disorders, dry eyes, and obesity.
3. It is desirable to keep DTx devices next to the sleeping area during sleep.	3. It is unnecessary to understand the culture of children's smartphone usage.
4. Engaging in offline activities with family is unnecessary for preventing children's overdependence on DTx.	4. It is desirable to set your child's smartphone rules in a coercive manner.
5. It is desirable not to use features or applications aimed at preventing overdependence on DTx as they may antagonize children.	5. It is beneficial to make time away from smartphone and spend time with family to prevent excessive use of smartphone.
6. The treatment plan of DTx for your child should be discussed at each routine clinic visit.	6. Children should be instructed not to cause any harm to others by using smartphones in public places.

[2] Children and Adolescents version

**Guideline A (Experimental group)**	**Guideline B (Control group)**
The following is information regarding the guidelines. Please read the sentences and mark O for correct and X for incorrect.	The following is information regarding the guidelines. Please read the sentences and mark O for correct and X for incorrect.
1. Overdependence on DTx can lead to negative symptoms such as back pain, dry eyes, and weight gain.	1. It is important to perform eye health exercises and stretching after using a smartphone.
2. DTx devices should only be used for the prescribed duration at a designated location.	2. It is better to specify smartphone usage time, such as '30 minutes' or '1 hour'.
3. It is desirable not to keep DTx devices right next to where you sleep.	3. Deleting apps that are not used can help prevent excessive use of smartphone
4. Physical activities for at least one hour daily is recommended to maintain a balance between online and offline life.	4. Unplanned smartphone usage can have a positive impact on preventing excessive use of smartphone.
5. It is better to use DTx rather than focusing on the conversation, when talking with family.	5. It is safer to hold the smartphone while walking rather than storing it in a bag.
6. It is better not to use features or applications aimed at preventing overdependence on DTx, as they may control me.	6. It is not necessary to set smartphones to silent or vibrate mode in quiet public places like libraries or movie theaters.

Figure S1 in Multimedia Appendix 1. Pie chart of the purpose for smartphone usage from children and adolescents on weekdays (A) and on weekends (B).

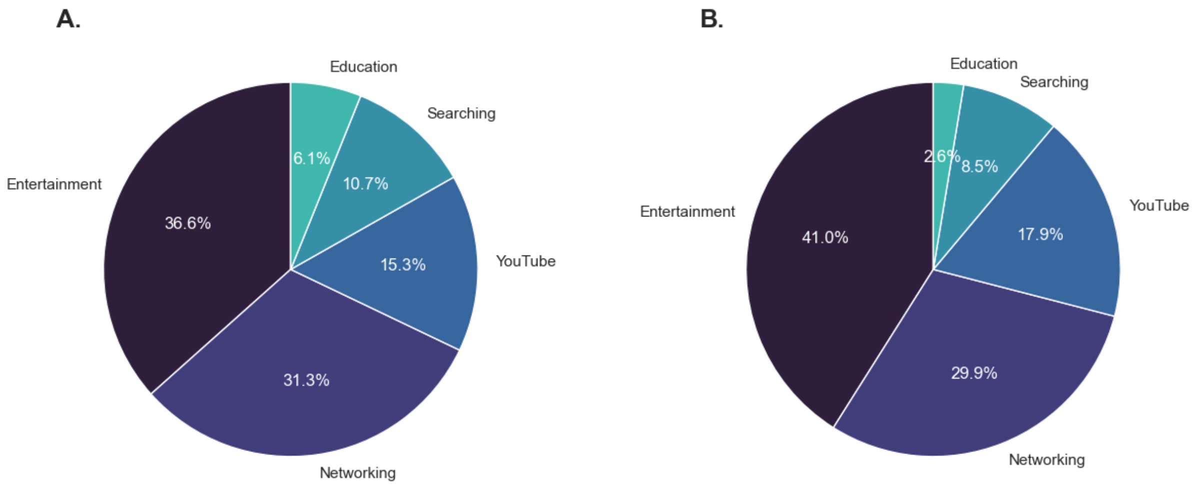

In Phase I, Children and Adolescents (ChAd) answered to the purpose of their smartphone usage through subjective responses. On weekdays, the predominant purpose of smartphone usage was entertainment (48/131 responses, 36.6%), followed by networking (41/131, 31.3%) and YouTube (20/131, 15.3%). On weekends, entertainment (48/117, 41%) remained the most predominant purpose, followed by networking (35/117, 29.9%) and YouTube (21/117, 17.9%). The primary purpose of smartphone usage was consistent on both weekdays and weekends, with entertainment, networking, and YouTube ranking in the same order. However, the difference between entertainment and networking on weekends was 11.1%P, while on weekdays, it was 5.3%P. Additionally, education on weekdays decreased by 3.5%P, from 6.1% to 2.6% on weekends.

Meanwhile, caregivers asked about their efforts being made to prevent smartphone addiction in children. Nineteen caregivers (38.8%) used children protection applications to prevent smartphone addiction, which is prevalent in the results. In addition, fifteen caregivers (30.6%) answered that they lead through conversation. Efforts to prevent smartphone addiction include encouraging children to self-regulate (6 individuals, 12.2%), diverting attention to other activities (5, 10.2%), returning smartphones before bedtime (3, 6.1%) and using medication (1, 2%). There were five caregivers who didn't make any efforts. All multiple responses were included in these results.

Table S3 in Multimedia Appendix 1. Subjective responses on Phase I survey for development of Guideline A.

Theme	Subtheme	Verbatim examples
**Caregiver**		
**Potential advantages of guidelines**	Prevention of side effects and overdependence	"With guideline, overdependence will be reduced."<br>"The concern about side effects will decrease without an increased risk of dependency."
	Enhancing children's self-regulation	"It will help my child develop patience for self-regulation."<br>"The child may receive assistance in areas where they are unable to self-regulate."
	Increased trust in treatment	"Given the provided guidelines, the use of DTx interventions seems to be safe and reliable."<br>"Parents may feel that DTx interventions have stability, if there is guideline."
	Reducing caregiver's anxiety	"Parents' anxiety about using the treatment seems to decrease"<br>"Since it is related to digital-related therapies, having guideline is essential, and it appears that they could be helpful for future child management."
	Motivation for treatment	"I think it will help with treatment by reducing resistance."<br>"The motivational aspect of embracing and actively engaging in the treatment positively."
**Potential disadvantages of guidelines**	Side effects and overdependence	"It seems there may be issues such as visual impairment."<br>"I am also worried about diseases such as cervical disc herniation."<br>"Negative response caused by DTx."
	Increased exposure time to digital devices	"Using digital devices for other purposes than the designated treatment during the intervention time."<br>"I am concerned that the use of DTx may lead to an increase in overall device usage, such as smartphones."
	Decreased effects due to non-individualized guideline	"While there may be differences in treatment progress for each child, it's uncertain whether the guideline will take all of that into consideration."<br>"Not autonomous guidelines, especially among adolescents, can lead to resistance towards interventions."
	Conflict with family members	"Conflict between parents and children may arise regarding DTx due to resistance or reluctance towards time limits and usage restrictions."<br>"I am worried that adolescents will follow the rules (guideline) well."
**Expectations for guidelines**	Parental monitoring	"Parents' continuous attention and monitoring will be helpful."<br>"It would better there be monitoring apps on parents' phones."
	Blocking other applications while using DTx	"Auto blocking function"<br>"Disable other functions when running DTx"

Theme	Subtheme	Verbatim examples
	Ongoing feedback from professionals	"Constant feedback from the child and medical staff will be needed."<br>"I think that DTx should be used under the guidance of experts."
	Concurrent use with other treatments	"Other treatments must be combined (drugs, doctor's appointment, etc.)"<br>"It would be good to have guidelines that can be applied in real life."
	Activities for relief	"It would be a good idea to allow time for stretching or (emotional) relaxation during treatment."
**Children and Adolescents**		
**Potential advantages of guidelines**	Prevention of side effects and overdependence	"It is expected to aid in overdependence management, leading to more efficient and safe use of DTx."<br>"It may be helpful in preventing overdependence."
	Enhancing self-regulation	"I can improve my ability to control usage time."<br>"I would enable more restrained usage with rules."
	Increased reliability of DTx	"Treatments with guidelines can be used with more trust and reliance."<br>"There will be no confusion as long as guidelines are provided."
	Timely feedback	"Feedback can be given and received smoothly, improving treatment effectiveness."<br>"It will be possible to more quickly determine the patient's condition, since digital devices are used in everyday life."
	Motivation for treatment	"Since it is a treatment that has guidelines applied, I think I would like to try using it more."
**Potential disadvantages of guidelines**	Side effects and overdependence	"It seems to be a risk of overdependence, because it is treatment with digital devices.",<br>"I might become addicted if I do it too much."
	Ignoring guideline	"People who do not adhere to the guidelines may not experience the desired effects."<br>"Even if there are guidelines, I am concerned that there are many cases where they are not followed."
	Setback in treatment due to non-individualized or strict guideline	"If the same guideline is provided to people in need of more treatment, it may be inadequate."<br>"The flow of using DTx suddenly ends at an important treatment point, treatment may be slowed or disrupted."
	Conflict with family members	"It could lead to conflicts within the family, if the rules are not followed."<br>"I'm worried that while I'm using DTx, my mom will come kicking in the room, yelling at me to study."

Note S1 in Multimedia Appendix 1. Guidelines provided for Phase II of this study. Guideline A is for the experimental group, and Guideline B is for the control group.

[1] Guideline A

Caregiver Guideline

CHECK : Monitoring Your Child's Digital Device Dependency

1. Examine your child's digital device usage habits.
   - Assess your child's condition using Smartphone Dependency Scale (SAS) and Internet Gaming Addiction Screening Tool (IGUESS) in the appendix.
   - If your child is **the high-risk or positive on one or both scales, caution is recommended** when using digital therapeutics.
2. Discuss the negative symptoms that may arise due to overuse of digital device with your child.
   - **Physical Health**: dry eyes, impaired vision, noise-induced hearing loss, "text neck" syndrome, carpal tunnel syndrome, musculoskeletal issues, spinal problems due to incorrect posture
   - **Activities**: overweight, obesity, reduced sleep duration, decreased outdoor and physical activities
   - **Mental Health**: anxiety, depression, excessive behaviors, increased aggression
   - **Interpersonal Relationships**: drifting away from real-life friends, conflicts with parents, maladjustment in school life

PLAN : Establishing the Digital Therapeutics Plan for Your Child

- For children aged 6-12, it is advisable to develop a digital therapy plan together, while for adolescents aged 13-18, it is preferable to guide them in creating their own plan.

3. Check the purpose and effectiveness of digital therapeutics with your child.
   - ✓ Device (Name): ____________________
   - ✓ Purpose: ____________________
   - ✓ Effect of Use: ____________________

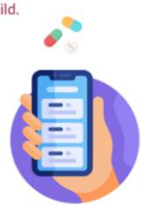

4. Reviewing your child's Digital Therapeutics Plan.
   - ✓ Duration: ____________ ~ ____________
   - ✓ Frequency: _______ times per day, _______ minutes per session
   - ✓ Primary Place of Use: ____________________

- Teach the use of digital therapeutics **only at prescribed times and specific places**.
- Display these guidelines **in a visible location that everyone can check**, such as a calendar, refrigerator, or living room wall.
- **Regularly review** the digital therapeutics plan with the primary physician during **scheduled medical appointments**.
- Encourage your child to **understand the purpose and method** of using digital therapeutics in advance so that they can adhere to the usage plan independently.

1 / 3

ACTION : Applying Digital Therapeutics Prevention in real life

4. Store digital therapeutic devises in a designated location when no in use.
   - Especially **during bedtime**, guide your child to place devices **at a distance from sleep area**. Keep them on a desk or in a drawer away from the bed to avoid disrupting sleep. The light, vibrations, and sounds from digital therapy devices at night can worsen the quality of sleep.
     - ✓ Storage Location: ____________________
6. In the morning before school and 1 hour before bedtime, guide your child to avoid using digital therapeutic.
   - It is advisable to guide your child not to use devices **between 6 AM and 9 AM** before going to school. Using digital screens in the morning can negatively impact your child's attention span and daytime fatigue.
     - ✓ School Hours: ____________________
     - ✓ Do not use digital therapeutics before the above time.
   - **From 10 PM to 2 AM at night** is the golden time for sleep. During this time, the secretion of growth hormones and melatonin (a hormone helping deep sleep) is promoted. The blue light emitted from digital screens can disrupt sleep cycles and have a negative impact on health.
     - ✓ Average Bedtime: ____________________
     - ✓ Don not use digital therapeutics 1 hour before the above time.
7. Help your child maintain a balance with offline activities.
   - Plan offline activities that involve the family. Children and adolescents require **at least 60 minutes of physical activity every day**. Encourage physical activities such as walks, exercise, dancing, and more, or engage in them together. Plan activities that take away from digital screens participate in the real world, such as local cultural programs, visiting libraries, watching performances, volunteering.
     - ✓ Write down offline activities you can do with your child.
       - ❑ Ex) Attend a performance with family once a month.
       - ❑ ____________________
       - ❑ ____________________

     TIPS Include specific dates, times and locations in plan. Ask your child what they want to do.
   - Have **Digital Therapeutics Free Time** everyday. Turn off digital devices during meal times, while walking the pet, or during car or bus rides, creating opportunities for meaningful conversations with family.
     - ✓ Write down activities your child can do during Digital Therapeutics Free Time.
       - ❑ Ex) Share daily routines with having dinner at least 5 times a week.
       - ❑ ____________________
       - ❑ ____________________

     TIPS Include details about the time and frequency. Discuss with your child when he/she can spend time for these activities.

2 / 3

8. When using digital therapies, make sure to prevent side effects through stretching and eye protection.
    - Alleviate muscles and ligament by stretching your neck, shoulders, waist, and wrists at least once every hour.
    - Take a 10-minute break every 50 minutes or allow your eyes to rest for about 20 seconds by closing them or looking at a distant object every 20 minutes.

SMART : Using Digital Therapeutics Wisely

9. Utilize functions or applications that can help prevent addiction on digital therapeutics.
    - **Automatic Shutdown**: automatically terminate usage after a specific time.
    - **Usage Alerts**: enable notifications or vibrations based on usage time, accessible to both the child and their caregivers.
    - **Blocking Other Apps**: block access or notifications from other applications while using digital therapeutics.
    - **Monitoring Application**: allows caregivers to monitor the child's use of digital therapeutics.
        - **Tracking Progress**: Track weekly goals, progress, and real-time data regarding usage time.

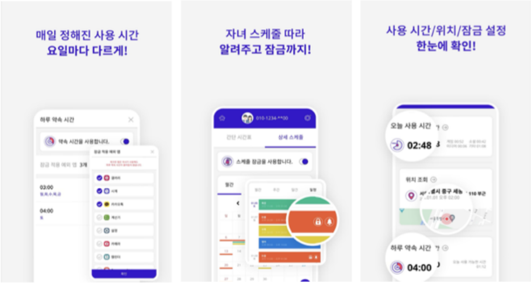

Example of a Monitoring Application (Source: App Store, T Youth Safety Pack)

TIPS

When using a monitoring application for caregivers, it's essential to go through a process of obtaining consent from your child based on sufficient explanation. Coercive parental control can create resistance and conflicts with child. Explain to child that you are using it to check their treatment progress and help them develop healthy digital therapeutic usage habits. Above all, remember to prioritize encouraging them to stick to their usage plan independently!

10. Regularly adjust the child's treatment plan through appointments with the primary physician (outpatient visits or contact).
    - Discuss your child's symptoms of addiction on digital therapeutics when consulting with professionals.
    - If you are having challenges guiding your child in their use of digital therapeutics, consider seeking professional advice.

Children Guideline (Ages 6-12)

CHECK : Monitoring My Digital Device Dependency

1. Examine your digital device usage habits with your parents.
    - Assess your condition using Smartphone Dependency Scale (SAS) and Internet Gaming Addiction Screening Tool (IGUESS) in the appendix.
    - If you are the **high-risk or positive on one or both scales, caution is recommended** when using digital therapeutics.

2. Discuss the negative symptoms that may arise due to overuse of digital device with your parents.
    - **Physical Health**: dry eyes, impaired vision, noise-induced hearing loss, "text neck" syndrome, carpal tunnel syndrome, musculoskeletal issues, spinal problems due to incorrect posture
    - **Activities**: overweight, obesity, reduced sleep duration, decreased outdoor and physical activities
    - **Mental Health**: anxiety, depression, excessive behaviors, increased aggression
    - **Interpersonal Relationships**: drifting away from real-life friends, conflicts with parents, maladjustment in school life

PLAN : Establishing the Digital Therapeutics Plan

3. Check the purpose and effectiveness of digital therapeutics with your parents.
    - ✓ Device (Name): ______________________________
    - ✓ Purpose: ______________________________
    - ✓ Effect of Use: ______________________________

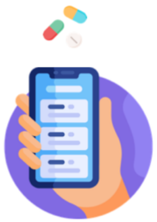

4. Plan the digital therapeutic plan with your parents.
    - ✓ Duration: ________________ ~ ________________
    - ✓ Frequency: _______ times per day, _______ minutes per session
    - ✓ Primary Place of Use: ______________________________

- Promise to use digital therapy only for **the prescribed duration at specific location**.
- Place these guidelines **in a visible location where everyone can check**, such as on the calendar, refrigerator, or living room wall.
- **Regularly review** your digital therapeutics plan with your doctor during **scheduled medical appointments**.
- It's important to understand the purpose of using digital therapeutics and to stick to your plan.

ACTION : Applying Digital Therapeutics Prevention in real life

5. Store digital therapeutic devises in a designated location when no in use.

- Especially **during sleep**, place devices **at a distance from your sleeping area**. Put them on a desk or drawer away from the bed to avoid disrupting your sleep. The light, vibrations, and sounds that may come from digital therapeutics during the night can worsen the quality of sleep.

  ✓ Storage Location: ____

6. In the morning before school and 1 hour before bedtime, avoid using digital therapeutics.

- Before going to school, it's best not to use digital **therapeutics between 6 AM and 9 AM**. Using digital screens in the morning can lower your concentration and make you tired during the day.

  ✓ School Hours: ________________________________

  ✓ Do not use digital therapeutics before the above time.

- **From 10 PM to 2 AM at night** is the golden time for sleep. During this time, the secretion of growth hormones and melatonin (a hormone helping deep sleep) is promoted. The blue light emitted from digital screens can disrupt the sleep cycle and negatively affect your health.

  ✓ Average Bedtime: ___________________________________

  ✓ Don not use digital therapeutics 1 hour before the above time.

7. Try maintaining a balance online and offline life.

- Participate in offline activities with your family. It is a good idea to engage in **physical activity for at least one hour every day**, such as walking, exercising, or dancing. Engaging in activities that take away from digital screens and participate in the real world, such as local programs, visiting libraries, watching performances, and volunteering.

  ✓ Write down offline activities that you can do.

  ❑ Ex) Attend a performance with family once a month.

  ❑ ____

  ❑ ____

  TIPS
  Include specific dates, times, and locations in plan. Talk your parents what you want to do.

- Have **Digital Therapeutics Free Time** everyday. When talking to family, such as while eating, walking your pet, or traveling in the car or bus, turn off your digital devices and focus more on the conversation.

  ✓ Write down activities you can do during Digital Therapeutics Free Time.

  ❑ Ex) Share daily routines with having dinner at least 5 times a week.

  ❑ ____

  ❑ ____

  TIPS
  Include details about time and frequency. Discuss with your parents when you can spend time for these activates.

8. Prevent side effects through stretching and eye protection when using digital therapeutics.

- Relieve muscles and ligaments in **your neck, shoulders, waist, and wrists by stretching** them **at least once an hour**.
- If you feel discomfort in your hands or neck while using digital therapeutics, stop using it **and take a 10-minute break**.
- Bending your head or waist to look down, lying down, or leaning on something puts a strain on your neck and shoulders. Use digital therapeutics in the correct posture, **keeping your head straight without bending,** and **maintaining a straight posture of your waist, aligning the screen with your eye level**.

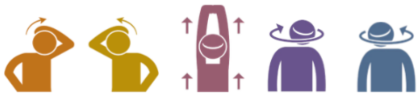

- Allow your eyes to rest for about 20 seconds every 20 minutes by closing your eyes or looking at a distant object.
- If the screen of device is too bright, it can easily dry out your eyes and lead to fatigue. **Adjust the screen brightness of device** and **keep a distance of at least 30 cm to protect your eyes**.

SMART : Using Digital Therapeutics Wisely

9. Utilize functions or applications that can help prevent addiction on digital therapeutics.

- **Automatic Shutdown**: automatically terminate usage after a specific time.
- **Usage Alerts**: enable notifications or vibrations based on usage time, accessible to both the child and their caregivers.
- **Blocking Other Apps**: block access or notifications from other applications while using digital therapeutics.

TIPS
When using digital therapeutics, it becomes difficult for anyone to adhere to self-imposed rules. In such cases, using functions or applications that can help prevent overdependence can make it easier to follow the rules. How about thinking of it not as something that controls you but as something that helps you?

9. Regularly adjust your treatment plan through appointments with your doctor.

- Talk to your doctor about your symptoms of addiction on digital therapeutics.
- If you are having challenges using digital therapeutics, feel free to request counseling at any time.

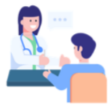

Adolescents Guideline (Ages 13-18)

CHECK : Monitoring My Digital Device Dependency

1. Examine your digital device usage habits with your parents.

- Assess your condition using Smartphone Dependency Scale (SAS) and Internet Gaming Addiction Screening Tool (IGUESS) in the appendix.
- If you are the **high-risk or positive on one or both scales, caution is recommended** when using digital therapeutics.

2. Discuss the negative symptoms that may arise due to overuse of digital device with your parents.

- **Physical Health**: dry eyes, impaired vision, noise-induced hearing loss, "text neck" syndrome, carpal tunnel syndrome, musculoskeletal issues, spinal problems due to incorrect posture
- **Activities**: overweight, obesity, reduced sleep duration, decreased outdoor and physical activities
- **Mental Health**: anxiety, depression, excessive behaviors, increased aggression
- **Interpersonal Relationships**: drifting away from real-life friends, conflicts with parents, maladjustment in school life

PLAN : Establishing the Digital Therapeutics Plan

3. Check the purpose and effectiveness of your digital therapeutics.

✓ Device (Name): ____________________

✓ Purpose: ____________________

✓ Effect of Use: ____________________

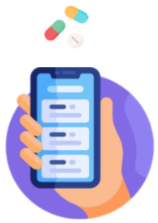

4. Plan your own the digital therapeutic plan.

✓ Duration: __________ ~ __________

✓ Frequency: _______ times per day, _______ minutes per session

✓ Primary Place of Use: ____________________

- Promise to use digital therapy **only for the prescribed duration at specific location**.
- Please place these guidelines **in a visible location where everyone can check**, such as on the calendar, refrigerator, or living room wall.
- **Regularly review** your digital therapeutics plan with your doctor during **scheduled medical appointments**.
- It's important to understand the purpose of using digital therapeutics and to stick to your plan.

ACTION : Applying Digital Therapeutics Prevention in real life

5. Store digital therapeutic devises in a designated location when no in use.

- Especially during sleep, place devices **at a distance from your sleeping area**. Put them on a desk or drawer away from the bed to avoid disrupting your sleep. The light, vibrations, and sounds that may come from digital therapeutics during the night can worsen the quality of sleep.

  ✓ Storage Location: ____________________

6. In the morning before school and 1 hour before bedtime, avoid using digital therapeutics.

- Before going to school, it's best not to use digital **therapeutics between 6 AM and 9 AM**. Using digital screens in the morning can lower your concentration and make you tired during the day.

  ✓ School Hours: ____________________

  ✓ Do not use digital therapeutics before the above time.

- **From 10 PM to 2 AM at night** is the golden time for sleep. During this time, the secretion of growth hormones and melatonin (a hormone helping deep sleep) is promoted. The blue light emitted from digital screens can disrupt the sleep cycle and negatively affect your health.

  ✓ Average Bedtime: ____________________

  ✓ Don not use digital therapeutics 1 hour before the above time.

7. Try maintaining a balance online and offline life.

- Participate in offline activities with your family or friends. It is a good idea to engage in **physical activity for at least one hour every day**, such as walking, exercising, or dancing. Engaging in activities that take away from digital screens and participate in the real world, such as local programs, visiting libraries, watching performances, and volunteering.

  ✓ Write down offline activities that you can do.

  ❑ Ex) Play badminton with friends every Saturday at 2pm

  ❑ ____________________

  ❑ ____________________

  TIPS
  Include specific dates, times, and locations in plan. Talk parents or friends what you want to do.

- Have **Digital Therapeutics Free Time** everyday. When talking to family or friends, such as while eating, walking your pet, or traveling in the car or bus, turn off your digital devices and focus more on the conversation.

  ✓ Write down activities you can do during Digital Therapeutics Free Time.

  ❑ Ex) Share daily routines with having dinner at least 5 times a week.

  ❑ ____________________

  ❑ ____________________

  TIPS
  Include details about time and frequency. Discuss with parents or friends when you can spend time for these activates.

8. Prevent side effects through stretching and eye protection when using digital therapeutics.

- Relieve muscles and ligaments in **your neck, shoulders, waist, and wrists by stretching** them **at least once an hour**.
- If you feel discomfort in your hands or neck while using digital therapeutics, stop using it **and take a 10-minute break**.
- Bending your head or waist to look down, lying down, or leaning on something puts a strain on your neck and shoulders. Use digital therapeutics in the correct posture, **keeping your head straight without bending,** and **maintaining a straight posture of your waist**, **aligning the screen with your eye level**.

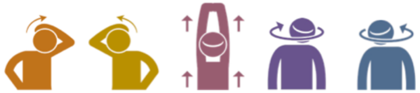

- Allow your eyes to rest for about 20 seconds every 20 minutes by closing your eyes or looking at a distant object.
- If the screen of device is too bright, it can easily dry out your eyes and lead to fatigue. **Adjust the screen brightness of device** and **keep a distance of at least 30 cm to protect your eyes**.

SMART : Using Digital Therapeutics Wisely

9. Utilize functions or applications that can help prevent addiction on digital therapeutics.

- **Automatic Shutdown**: automatically terminate usage after a specific time.
- **Usage Alerts**: enable notifications or vibrations based on usage time, accessible to both the child and their caregivers.
- **Blocking Other Apps**: block access or notifications from other applications while using digital therapeutics.

TIPS

When using digital therapeutics, it becomes difficult for anyone to adhere to self-imposed rules. In such cases, using functions or applications that can help prevent overdependence can make it easier to follow the rules. How about thinking of it not as something that controls you but as something that helps you?

9. Regularly adjust your treatment plan through appointments with your doctor.

- Talk to your doctor about your symptoms of addiction on digital therapeutics.
- If you are having challenges using digital therapeutics, feel free to request counseling at any time.

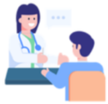

Smartphone Addiction Scale (SAS)

Please respond to the following questions regarding your child's smartphone usage.

	Items	Strong disagreement	Disagreement	Agreement	Strong agreement
1	It fails every time you reduce the smartphone hours.	1	2	3	4
2	It is difficult to control the usage time of smartphone.	1	2	3	4
3	It is difficult to keep proper smartphone usage time.	1	2	3	4
4	It is hard to focus on other things if you have a smartphone next to you.	1	2	3	4
5	Thinking of smartphone does not leave your head.	1	2	3	4
6	I strongly desire to use smartphone.	1	2	3	4
7	I have problems with my health because of smartphone use.	1	2	3	4
8	I have a hard time with my family because of smartphone use.	1	2	3	4
9	Due to the use of smartphones, I have experienced severe conflicts in friends, colleagues, and social relationships.	1	2	3	4
10	Due to the smartphone, there are difficulties in performing tasks (such as study or work).	1	2	3	4
				Total Score (	)

1 (Strong disagreement) - 4 (Strong agreement)

Scores for each item are summed to calculate the total score, with a total score range of 10-40 points.

- **High-Risk Group (31 points and above)**

This indicates a state where control over smartphone use is lost, leading to serious issues such as interpersonal conflicts or disruptions in daily roles.

- **Potential Risk Group (30 points or below to 23 points and above)**

This suggests a weakened ability to control smartphone use, resulting in increased usage leading to emerging problems in daily life.

- **General User (22 points or below)**

Internet Game Use-Elicited Symptom Screen (IGUESS)

Please respond to the following questions regarding your child's gaming habits.

	Over the past 12 months, how much have you experienced the following symptoms related to online gaming?※	Not at all	Rarely	Some times	Always
1	Have you spent a lot of time thinking about games or planned gaming?	0	1	2	3
2	Did you get annoyed, uneasy or upset when you couldn't play?	0	1	2	3
3	Have you felt the need to play more and more?	0	1	2	3
4	Have you tried to cut down on gaming without succeeding?	0	1	2	3
5	Have you lost interest in previous hobbies and leisure activities because of gaming?	0	1	2	3
6	Did you continue to play even though it created problems for you?	0	1	2	3
7	Have you lied to family members, therapists or others about how much you have played?	0	1	2	3
8	Did you play to reduce negative feelings (like helplessness, guilt, anxiety)?	0	1	2	3
9	Have you risked or ruined an important relationship, job, education or career opportunity because of gaming?	0	1	2	3
				Total Score (	)

0 (Not at all) - 3 (Always)

Scores for each item are summed to calculate the total score, with a total score range of 0-27 points.

- Positive Diagnosis Criteria: 10 points or above

This indicates a high-risk group for internet gaming disorder. It is necessary to confirm the diagnosis of internet gaming disorder through consultation with a professional.

❖ This assessment tool is a screening tool. Therefore, caution should be exercised not to mistake a positive diagnosis on the test result as a confirmed diagnosis of internet gaming disorder.

Smartphone Addiction Scale (SAS)

Please respond to the following questions regarding your own smartphone usage.

	Items	Strong disagreement	Disagreement	Agreement	Strong agreement
1	It fails every time you reduce the smartphone hours.	1	2	3	4
2	It is difficult to control the usage time of smartphone.	1	2	3	4
3	It is difficult to keep proper smartphone usage time.	1	2	3	4
4	It is hard to focus on other things if you have a smartphone next to you.	1	2	3	4
5	Thinking of smartphone does not leave your head.	1	2	3	4
6	I strongly desire to use smartphone.	1	2	3	4
7	I have problems with my health because of smartphone use.	1	2	3	4
8	I have a hard time with my family because of smartphone use.	1	2	3	4
9	Due to the use of smartphones, I have experienced severe conflicts in friends, colleagues, and social relationships.	1	2	3	4
10	Due to the smartphone, there are difficulties in performing tasks (such as study or work).	1	2	3	4
				Total Score (	)

1 (Strong disagreement) - 4 (Strong agreement)

Scores for each item are summed to calculate the total score, with a total score range of 10-40 points.

- **High-Risk Group (31 points and above)**

This indicates a state where control over smartphone use is lost, leading to serious issues such as interpersonal conflicts or disruptions in daily roles.

- **Potential Risk Group (30 points or below to 23 points and above)**

This suggests a weakened ability to control smartphone use, resulting in increased usage leading to emerging problems in daily life.

- **General User (22 points or below)**

Internet Game Use-Elicited Symptom Screen (IGUESS)

Please respond to the following questions regarding your own gaming habits.

	Over the past 12 months, how much have you experienced the following symptoms related to online gaming?	Not at all	Rarely	Some times	Always
1	Have you spent a lot of time thinking about games or planned gaming?	0	1	2	3
2	Did you get annoyed, uneasy or upset when you couldn't play?	0	1	2	3
3	Have you felt the need to play more and more?	0	1	2	3
4	Have you tried to cut down on gaming without succeeding?	0	1	2	3
5	Have you lost interest in previous hobbies and leisure activities because of gaming?	0	1	2	3
6	Did you continue to play even though it created problems for you?	0	1	2	3
7	Have you lied to family members, therapists or others about how much you have played?	0	1	2	3
8	Did you play to reduce negative feelings (like helplessness, guilt, anxiety)?	0	1	2	3
9	Have you risked or ruined an important relationship, job, education or career opportunity because of gaming?	0	1	2	3
			Total Score (		)

0 (Not at all) - 3 (Always)

Scores for each item are summed to calculate the total score, with a total score range of 0-27 points.

- Positive Diagnosis Criteria: 10 points or above

This indicates a high-risk group for internet gaming disorder. It is necessary to confirm the diagnosis of internet gaming disorder through consultation with a professional.

❖ This assessment tool is a screening tool. Therefore, caution should be exercised not to mistake a positive diagnosis on the test result as a confirmed diagnosis of internet gaming disorder.

[2] Guideline B

Caregiver Guideline

R (Step 1)

Recognizing Smartphone Addiction Issues - PROBLEM **R**ECOGNITION

What problems can arise from excessive smartphone usage?

1. Discuss the potential **negative symptoms** of excessive smartphone usage.
   - Excessive smartphone use can have negative impacts on physical health and interpersonal relationships.
     - **Physical Health**: dry eyes, impaired vision, noise-induced hearing loss, text neck syndrome, carpal tunnel syndrome, musculoskeletal issues, spinal problems due to incorrect posture
     - **Activities**: overweight, obesity, reduced sleep duration, decreased outdoor and physical activities
     - **Mental Health**: anxiety, depression, excessive behaviors, increased aggression
     - **Interpersonal Relationships**: drifting away from real-life friends, conflicts with parents, maladjustment in school life
   - Improper posture and usage habits can lead to health issues.

E (Step 2)

Check Smartphone Usage Status - STATE CHECK

How is your child's smartphone addiction? Check it out!

2. Assess if your child is **using smartphone excessively**.
   - Utilize the Smartphone Addiction Scale (SAS) to determine if your child might be dependent on smartphone.

	Items	Strong disagreement	Disagreement	Agreement	Strong agreement
1	It fails every time you reduce the smartphone hours.	1	2	3	4
2	It is difficult to control the usage time of smartphone.	1	2	3	4
3	It is difficult to keep proper smartphone usage time.	1	2	3	4
4	It is hard to focus on other things if you have a smartphone next to you.	1	2	3	4
5	Thinking of smartphone does not leave your head.	1	2	3	4
6	I strongly desire to use smartphone.	1	2	3	4
7	I have problems with my health because of smartphone use.	1	2	3	4
8	I have a hard time with my family because of smartphone use.	1	2	3	4
9	Due to the use of smartphones, I have experienced severe conflicts in friends, colleagues, and social relationships.	1	2	3	4
10	Due to the smartphone, there are difficulties in performing tasks (such as study or work).	1	2	3	4
				Total Score (	)

1 (Strong disagreement) - 4 (Strong agreement)

Scores for each item are summed to calculate the total score, with a total score range of 10-40 points.

- **High-Risk Group (31 points and above)**

This indicates a state where control over smartphone use is lost, leading to serious issues such as interpersonal conflicts or disruptions in daily roles.

- **Potential Risk Group (30 points or below to 23 points and above)**

This suggests a weakened ability to control smartphone use, resulting in increased usage leading to emerging problems in daily life.

- **General User (22 points or below)**

S (Step 3)

Practical Strategies for Smartphone Usage - **S**UGGEST AN ALTERNATIVE

What can you do you help your child use smartphone properly?

3. Understand your child's smartphone usage culture and guide them toward responsible use.
   - Express interest in your child's favorite smartphone activities or apps and use them as tools for conversation.
   - Explore applications that can benefit your child's school life, friendships, and stress management, and discuss how to use them appropriately.
   - Find useful apps related to your child's interests or academics and use them together as a means of communication.

4. Provide **sleep guidance** to your childe who struggle with sleeping problem.
   - Use timers and alarms to help them establish a bedtime routine and fall asleep at designated times.

5. Encourage your child to **set their own** smartphone usage rules.
   - Talk enough with child about smartphone usage and establish practical rules.
   - Try that everyone in the family is aware of and stick to smartphone usage rules.
   - Encourage child to turn off their smartphone not through coercion.

T (Step 4)

Enhancing Relationships with family and Friends CONNEC**T**

Keep distance from smartphone being with family and friends.

6. Have meal times with family and **talk** about topic you can relate to.
   - During meals, try talking about topics of interest your child, such as entertainment news or music.

7. **Engage in activities** like walks or watching performances that bring you closer to your child.
   - Participating in various activities together allows you to share your child's hobbies and interests, fostering communication.

8. (While walking/moving) Guide your child to **store** their smartphone **in their bag**.
   - Encourage the habit of putting the smartphone in their bag when walking or moving.

9. (In public space) Teach your child **basic etiquette** in public spaces like library or theater.
   - Set the phone to silent or vibrate mode and make an effort not to disturb others when making calls, listening to music, or watching videos.

Children Guideline (Ages 6-12)

R
(Step 1)

Recognizing Smartphone Addiction Issues - PROBLEM RECOGNITION

What problems can arise from excessive smartphone usage?

1. Excessive smartphone usage can **strain your body**.
   - After using for a certain period time, do eyes health exercise and stretching.
     - Eye Health Exercise

     [Eye movements - Up, Down, Left, Right]
     When your eyes are closed and then opened, move your eyes in the following sequence: up, down, right, left.

     [Eye movements - Upper Right, Lower Left, Upper Left, Lower Right]]
     Close your eyes and then open them, moving your eyes in the following sequence: upper right, lower left, upper left, lower right.

E
(Step 2)

Check Smartphone Usage Status - STATE CHECK

How is your smartphone addiction? Check it out for yourself!

2. Assess if you are using your smartphone excessively.
   - Utilize the "Smartphone Addiction Scale (SAS)" to determine if you might be dependent on your smartphone.

	Items	Strong disagreement	Disagreement	Agreement	Strong agreement
1	It fails every time you reduce the smartphone hours.	1	2	3	4
2	It is difficult to control the usage time of smartphone.	1	2	3	4
3	It is difficult to keep proper smartphone usage time.	1	2	3	4
4	It is hard to focus on other things if you have a smartphone next to you.	1	2	3	4
5	Thinking of smartphone does not leave your head.	1	2	3	4
6	I strongly desire to use smartphone.	1	2	3	4
7	I have problems with my health because of smartphone use.	1	2	3	4
8	I have a hard time with my family because of smartphone use.	1	2	3	4
9	Due to the use of smartphones, I have experienced severe conflicts in friends, colleagues, and social relationships.	1	2	3	4
10	Due to the smartphone, there are difficulties in performing tasks (such as study or work).	1	2	3	4
				Total Score (	)

1 (Strong disagreement) - 4 (Strong agreement)
Scores for each item are summed to calculate the total score, with a total score range of 10-40 points.

- **High-Risk Group (31 points and above)**

This indicates a state where control over smartphone use is lost, leading to serious issues such as interpersonal conflicts or disruptions in daily roles.

- **Potential Risk Group (30 points or below to 23 points and above)**

This suggests a weakened ability to control smartphone use, resulting in increased usage leading to emerging problems in daily life.

- **General User (22 points or below)**

S
(Step 3)

Practical Strategies for Smartphone Usage - SUGGEST AN ALTERNATIVE

What can you do for using smartphone properly?

3. Set **specific usage limits** and make an effort to stop yourself.
   - Rather than saying, "Just a moment!" be more specific and say, "I'll use it for only 10 minutes!" Set clear goals and make an effort to control your usage time.

4. Install useful applications and **delete unnecessary applications**.
   - Explore apps that help you manage your smartphone usage effectively.
   - Delete apps that you haven't used in the past month.

5. Above all, it is important to make an effort to **keep your promises repeatedly**.
   - Clearly define your reasons and goals for smartphone usage, which will help you keep your promises.
   - Avoid unplanned or unnecessary smartphone use.

T
(Step 4)

Enhancing Relationships with family and Friends CONNECT

Keep distance from smartphone being with family and friends.

6. Establish **smartphone usage rules** for the sake of a healthy family.
   - Not to bring smartphones to family gatherings, such as meal table and living room.
   - Make specific rules with your parents, such as planning the time to use smartphones.

7. (While walking/moving) Always **store** your smartphone **in your bag**.
   - Make sure to keep your smartphone in your bag, when walking.

8. (In public space) Keep **basic etiquette** in place such as library or theater.
   - Set your phone to silent or vibrate mode and make an effort not to disturb others when making calls, listening to music, or watching videos.

Adolescents Guideline (Ages 13-18)

R
(Step 1)

Recognizing Smartphone Addiction Issues - PROBLEM **R**ECOGNITION

What problems can occur from using smartphone too much?

1. **Right posture** is better than comfortable.
   - Do not rest chin on your hand. Keep your back straight and maintain right posture.
   - After using for a certain period time, do eyes health exercise and stretching.
     - Eye Health Exercise

     [Eye movements - Up, Down, Left, Right]

     When your eyes are closed and then opened, move your eyes in the following sequence: up, down, right, left.

     [Eye movements - Upper Right, Lower Left, Upper Left, Lower Right]]

     Close your eyes and then open them, moving your eyes in the following sequence: upper right, lower left, upper left, lower right.

E
(Step 2)

Check Smartphone Usage Status - STAT**E** CHECK

How is your smartphone addiction? Check it out for yourself!

2. Check if you are using your smartphone too much.
   - Think about whether you have experienced a lack of concentration due to insufficient sleep or if you have had arguments with your family.
   - Check yourself if you are in high-risk group using Smartphone Addiction Scale.

	Items	Strong disagreement	Disagreement	Agreement	Strong agreement
1	It fails every time you reduce the smartphone hours.	1	2	3	4
2	It is difficult to control the usage time of smartphone.	1	2	3	4
3	It is difficult to keep proper smartphone usage time.	1	2	3	4
4	It is hard to focus on other things if you have a smartphone next to you.	1	2	3	4
5	Thinking of smartphone does not leave your head.	1	2	3	4
6	I strongly desire to use smartphone.	1	2	3	4
7	I have problems with my health because of smartphone use.	1	2	3	4
8	I have a hard time with my family because of smartphone use.	1	2	3	4
9	Due to the use of smartphones, I have experienced severe conflicts in friends, colleagues, and social relationships.	1	2	3	4
10	Due to the smartphone, there are difficulties in performing tasks (such as study or work).	1	2	3	4
				Total Score (	)

1 (Strong disagreement) - 4 (Strong agreement)

Scores for each item are summed to calculate the total score, with a total score range of 10-40 points.

- **High-Risk Group (31 points and above)**

This indicates a state where control over smartphone use is lost, leading to serious issues such as interpersonal conflicts or disruptions in daily roles.

- **Potential Risk Group (30 points or below to 23 points and above)**

This suggests a weakened ability to control smartphone use, resulting in increased usage leading to emerging problems in daily life.

- **General User (22 points or below)**

S
(Step 3)

Practical Strategies for Smartphone Usage - **S**UGGEST AN ALTERNATIVE

What can you do for using smartphone properly?

3. Set a shutdown time alarm and protect your **golden sleep time**.
   - To maintain a healthy lifestyle, set a shutdown time for your smartphone to ensure you have enough sleep.

4. Try a **Messenger Diet** for your smartphone.
   - Use messaging apps only when necessary and turn off notifications or using silent mode when not needed.
   - Delete apps that you haven't used in the past month.

5. Try using **useful apps** for your career and education.
   - Utilize your smartphone as a smart tool for self-management.

6. Get into the habit of keeping your smartphone in **a specific place**.
   - Decide on a specific place for storing your smartphone with your family.
   - Make an effort not to bring digital devices and chargers into the bedroom or bed.

T
(Step 4)

Enhancing Relationships with family and Friends CONNEC**T**

Keep distance from smartphone being with family and friends.

7. Try having a meal with your family and engage in **face-to-face** conversations with **eye contact**.
   - During meals, have relaxed conversations and avoid placing your smartphone on the dining table.

8. Follow **the rules** that set with our friend at school.
   - Develop a habit of turning off your smartphone during class.

9. (While walking/moving) Always **store** your smartphone **in your bag**.
   - Make sure to keep your smartphone in your bag, when walking.

10. (In public space) Keep **basic etiquette** in place such as library or theater.
   - Set your phone to silent or vibrate mode and make an effort not to disturb others when making calls, listening to music, or watching videos.
